# Prevalence of cardiac dyssynchrony and correlation with atrio-ventricular block and QRS width in dilated cardiomyopathy: an echocardiographic study

**DOI:** 10.5830/CVJA-2012-032

**Published:** 2012-08

**Authors:** JB Anzouan-Kacou, MP Ncho-Mottoh, C Konin, AR N’Guetta, KA Ekou, BJ Koffi, KE Soya, ME Tano, R Abouo-N’Dori

**Affiliations:** Institute of Cardiology of Abidjan, Abidjan, Ivory Coast; Institute of Cardiology of Abidjan, Abidjan, Ivory Coast; Institute of Cardiology of Abidjan, Abidjan, Ivory Coast; Institute of Cardiology of Abidjan, Abidjan, Ivory Coast; Institute of Cardiology of Abidjan, Abidjan, Ivory Coast; Institute of Cardiology of Abidjan, Abidjan, Ivory Coast; Institute of Cardiology of Abidjan, Abidjan, Ivory Coast; Institute of Cardiology of Abidjan, Abidjan, Ivory Coast; Institute of Cardiology of Abidjan, Abidjan, Ivory Coast

**Keywords:** heart failure, cardiomyopathy, dyssynchrony, echocardiography, Africa

## Abstract

**Introduction:**

Cardiac dyssynchrony causes disorganised cardiac contraction, delayed wall contraction and reduced pumping efficiency. We aimed to assess the prevalence of different types of dyssynchrony in patients with dilated cardiomyopathy (DCM), and to establish the correlation between atrio-ventricular block and atrio-ventricular dyssynchrony (AVD), and between impaired intra-ventricular conduction and the existence of inter-ventricular dyssynchrony (inter-VD) and intra-left ventricular dyssynchrony (intra-LVD).

**Methods:**

We included 40 patients in New York Heart Association stage III or IV, admitted consecutively with DCM with severe left ventricular dysfunction (left ventricular end-diastolic diameter ≥ 60 mm and/or ≥ 30 mm/m^2^) and left ventricular ejection fraction < 35%. Electrocardiographic and echocardiographic data were evaluated in all patients. Patients were divided into two groups: group 1: eight patients, with a QRS duration ≥ 120 ms, and all presented with left bundle branch block; group 2: 32 patients with a narrow QRS < 120 ms.

**Results:**

Overall, the mean age was 54.7 ± 16.8 years and patients in group 1 were older (67.2 ± 13.6 vs 51.5 ± 15.8 years, *p* = 0.01). The prevalence of atrio-ventricular dyssynchrony (AVD), inter-VD and intra-LVD was respectively 40, 47.5 and 70%. Two patients (5%) did not exhibit dyssynchrony. AVD was present with a similar frequency in the two groups (37.5% in group 1 vs 40.6% in group 2, *p* = 0.8). There was no correlation of the magnitude of AVD with the duration of the PR interval (from the beginning of the P wave to the beginning of the QRS complex) (*r*^2^ = 0.02, *p* = 0.37) or the QRS width (*r*^2^ = 0.01, *p* = 0.38). A greater proportion of patients with inter-VD was observed in group 1 (87.5 vs 60%, *p* = 0.03). There was a trend towards a more important inter-ventricular mechanical delay according to QRS width (*r*^2^ = 0.009, *p* = 0.06). The proportion of intra-LVD was similar in all groups, with a high prevalence (87.5% in group 1 and 65.6% in group 2, *p* = 0.39).

**Conclusion:**

The assessment of cardiac dyssynchrony is possible in our country. Intra-ventricular mechanical dyssynchrony had a high prevalence in patients with DCM, irrespective of the QRS width. These data emphasise the usefulness of echocardiography in the screening of patients.

## Abstract

In dilated cardiomyopathy (DCM), alterations in cardiac structure and function result in regions of early and late contraction, known as dyssynchrony.^1^ This dyssynchrony disorganises cardiac contraction, delays wall contraction and reduces pumping efficiency.^1^,^2^ Morbidity and mortality rates are higher in patients with severe left ventricular systolic dysfunction and ECG-derived prolonged QRS interval than in those with normal QRS duration.^3^

Three types of dyssynchrony may occur: atrio-ventricular dyssynchrony (AVD) with a discordance of contraction between the atria and ventricles, inter-ventricular dyssynchrony (inter-VD) with a discordance between the time of left and right ventricle contractions, and intra-left ventricular dyssynchrony (intra-LVD) with a discordance in the contraction of the walls of the left ventricle. A large number of studies have used echocardiography to assess dyssynchrony.^1^,^4^

Intra-LVD appears to be the principal factor associated with contractile impairment and is affected by cardiac resynchronisation therapy (CRT). CRT has been proven to reduce symptoms and hospitalisation for heart failure, and to improve quality of life, exercise capacity and the overall prognosis.^1^,^4^,^5^ In this study, we aimed to assess the prevalence of different types of dyssynchrony in patients with dilated cardiomyopathy, and to establish the correlation between atrio-ventricular block and AVD, and between impaired intra-ventricular conduction and the existence of inter-VD and intra-LVD.

## Methods

This prospective study was conducted at the Institute of Cardiology of Abidjan (ICA) from June to December 2009. We included patients in New York Heart Association (NYHA) stage III or IV, admitted consecutively with the following features: dilated cardiomyopathy with severe left ventricular dysfunction (left ventricular end-diastolic diameter > 60 mm and/or 30 mm/m^²^) as measured by M-mode echocardiography, and an ejection fraction < 35%, (by Simpson’s method). A total of 40 patients were included. We excluded patients with non-sinus rhythm or valvular heart disease.

Clinical and echocardiographic characteristics of the patients are shown in Table 1. All patients underwent a standard 12-lead electrocardiogram and an ultrasound examination, which included a specific evaluation of atrio-ventricular, inter-ventricular and intra-ventricular dyssynchrony. Examinations (M mode, two dimensional and Doppler evaluation) were performed using ultrasonographic equipment (General Electric VIVID 7 PRO).

**Table 1.** Clinical And Echocardiographic Characteristics Of The Patients

	*All patients (n = 40)*	*Group 1 (QRS ≥ 120 ms) (n = 8 patients)*	*Group 2 (QRS < 120 ms) (n = 32 patients)*	p*-value (group 1 vs group 2)*
Age (years)	54.7 ± 16.8	67.2 ± 13.6	51.5 ± 15.8	**0.01**
Males (*n*, %)	23 (57.5)	5 (62.5)	18 (56.3)	0.9
NYHA class III (*n*, %)	26 (65)	6 (75)	20 (62.5)	0.8
NYHA class IV (*n*, %)	14 (35%)	2 (25)	12 (37.5)	0.8
QRS width (ms)	100 ± 32.9	157.5 ± 29.1	85.6 ± 10.4	**0.03**
PR interval (ms)	180.7 ± 31.4	180 ± 4	180.9 ± 29.2	0.94
LVEF (%)	29 ± 5	31.3 ± 3.3	28 ± 5	0.14
LVEDD (mm)	66.7 ± 6.6	67.9 ± 6.1	66.4 ± 6.8	0.58
LVESD (mm)	57.7 ± 6.2	58.8 ± 5.2	57.3 ± 6.5	0.55
E/A	1.5 ± 1.1	1.7 ± 1	1.9 ± 0.9	0.64
DT (ms)	116 ± 5	125.3 ± 42.7	114.3 ± 52.3	0.6
Mitral regurgitation (degree)	1.5 ± 0.5	1.6 ± 0.5	1.5 ± 0.5	0.64
PASP (mmHg)	42.9 ± 9	43.3 ± 17.5	42.8 ± 15.3	0.93
SDTI	11.4 ± 2.5	11.6 ± 1.2	11.4 ± 2.7	0.58
LVFT/RR (%)	40.3 ± 8.3	42.3 ± 11.8	39.8 ± 7.3	0.45
IVMD (ms)	38.6 ± 19.3	54.4 ± 10.8	36.2 ± 18.7	**0.01**
ESD (ms)	66.5 ± 46.9	75.6 ± 28.9	64.2 ± 50.6	0.54

NYHA: New York Heart Association; LVEF: left ventricular ejection fraction; LVEDD: left ventricular end-diastolic diameter; LVESD: left ventricular end-systolic diameter; PASP: pulmonary artery systolic pressure; E/A: ratio of E wave (early filling) and A wave (atrial contraction) of mitral valve inflow in pulsed Doppler; DT: deceleration time of early filling velocity; SDTI: right ventricular S peak in tissue Doppler imaging; LVFT/RR: left ventricular filling time to cardiac cycle; IVMD: inter-ventricular mechanical delay; ESD: electrosystolic delay; the bold emphasis marks the significant differences with *p*-value < 0.05.

According to QRS duration, the patients were divided into two groups. Group 1 included eight patients with a QRS duration ≥ 120 ms and all presented with a left bundle branch block (LBBB). Group 2 included 32 patients with a narrow QRS < 120 ms.

Assessment of AVD: parameters collected included the duration of transmitral pulsed Doppler flow (LVFT) and the length of the cardiac cycle (RR). The mean left ventricular filling time to cardiac cycle (LVFT/RR × 100 < 40%) defined atrio-ventricular dyssynchrony.

Assessment of inter-VD: the pulmonary pre-ejection time (PPET) was measured from the beginning of the QRS complex to the beginning of the pulmonary flow velocity curve, recorded by pulse-wave (PW) Doppler in the left parasternal short-axis view. The aortic pre-ejection time (APET) was measured from the beginning of the QRS complex to the beginning of the aortic flow velocity curve, recorded by PW Doppler in the apical five-chamber view. The difference between the two values determined the inter-ventricular mechanical delay (IVMD); an IVMD > 40 ms was considered as the cut-off value for inter-ventricular dyssynchrony.

Assessment of intra-LVD: we determined by pulsed tissue Doppler the electrosystolic delay (ESD) from the onset of the QRS complex to the positive peak systolic (Sa) velocity for four basal segments (septal, lateral, inferior and anterior). Intra-LVD was considered to be present if one or more absolute differences (ΔS) > 40 ms were found between two segments.

## Statistical analysis

Data were collected and analysed with the software EPI INFO version 6.04 and Microsoft Excell 2010. Continuous variables were presented as mean ± standard deviation. Statistical analysis was performed with Fischer’s exact test for a significance level of 5% for the comparison of proportions. Comparisons between frequencies in the two groups were performed using the Chi-square test. Correlations between electrocardiographic and echocardiographic parameters were explored using Pearson’s correlation.

## Results

Overall, the mean age was 54.7 ± 168 years, and patients with QRS ≥ 120 ms were older than those with narrow QRS (67.2 ± 13.6 vs 51.5 ± 15.8 ms, *p* = 0.01) (Table 1). Most patients were male (57.5%). Systolic and diastolic left ventricular dysfunction, left ventricular end-diastolic dysfunction (LVEDD), systolic right ventricular dysfunction, and degree of mitral regurgitation were similar in the two groups. AVD was present in 16 patients (40%), inter-VD in 19 patients (47.5%) and intra-LVD in 28 patients (70%). Two patients (5%) did not exhibit dyssynchrony.

AVD alone was present in two patients (5%), inter-VD alone in five patients (12.5%) and intra-LVD alone in 10 patients (25%). Four patients (10%) had at the same time AVD, inter-VD and intra-LVD. AVD was present with a similar frequency in the two groups (37.5% in group 1 vs 40.6% in group 2, *p* = 0.8) and the duration of LV filling time to cardiac cycle showed no difference between the two groups (Table 1).

In the sub-group of 16 patients with AVD, the mean left ventricular filling time to cardiac cycle was 32.6 ± 3.9%. Six patients had a first-degree atrio-ventricular block (AVB1). There was no relationship between the existence of an AVB1 and AVD (Table 2). There was no correlation between the magnitude of AVD and the duration of the PR interval (from the beginning of the P wave to the beginning of the QRS complex) (*r*^²^ = 0.02, *p* = 0.37) or the QRS width (*r*^²^ = 0.01, *p* = 0.38).

**Table 2.** Relationship Between First-Degree Atrioventricular Block And Atrioventricular Dyssynchrony (*p* = 0.85)

	*Patients with AVB1*	*Patients without AVB1*	*Total*
Patients with AVD	4	12	16
Patients without AVD	2	22	24
Total	6	34	40

Mean IVMD was 38.6 ± 19.3 ms. The IVMD was significantly higher in patients from group 1 than in group 2 (54.4 ± 10.8 vs 36.2 ± 18.7 ms, *p* = 0.01) (Table 1). Inter-VD was present in 19 patients (47.5%) with a mean IVMD of 55.8 ± 10.6 ms. A greater proportion of patients with inter-VD was observed in group 1 (QRS ≥ 120 ms) compared to those in group 2 (87.5 vs 60%, *p* = 0.03). There was a trend of increasing importance of IVMD according to QRS width (*r*^2^ = 0.09, *p* = 0.06).

The mean ΔS was 66.5 ± 46.9 ms. The ESD was similar in the two groups. Intra-LVD was present in 28 patients (70%) with a mean ΔS of 84.8 ± 46.2 ms. The proportion of intra-LVD was similar in all groups, 87.5% in group 1 and 65.6% in group 2 (Table 3). In patients with intra-LVD, seven had an ESD ≥ 100 ms (17.5% of the total population and 25% of the patients with intra-LVD). Three of these seven patients had a QRS duration ≥ 120 ms.

**Table 3.** Relationship Between QRS Duration And Intraventricular Dyssynchrony (*p* = 0.39)

	*Group 1 (QRS ≥ 120 ms)*	*Group 2 (QRS < 120 ms)*	*Total*
Patients with intra-LVD	7	21	28
Patients without intra-LVD	1	11	12
Total	8	32	40

## Discussion

The study shows that in dilated cardiomyopathy, NYHA III to IV, cardiac dyssynchrony is common, regardless of QRS duration. Only two patients (5%) did not exhibit dyssynchrony. Intra-LVD seemed to be the most frequent type of mechanical dyssynchrony. The prevalence of AVD was 40%. There was no increase in this prevalence in the presence of AVB1.

Jurcut *et al*.^6^ found the prevalence of AVD was 45% among 58 patients hospitalised for DCM of different aetiologies. Abnormal conduction of the atrio-ventricular node resulted in delay between the atrial and ventricular contractions. Too much delay means that the valve leaflets are open in the mid-plane position as ventricular systole starts, resulting in pre-systolic mitral and tricuspid regurgitation. Prolonged atrio-ventricular delay also means that the diastolic filling period is abbreviated, limiting diastolic volume.^7^ In the CARE-HF study,^8^ prolonged PR interval and right bundle branch block (RBBB) were predictors of favourable outcome in patients with NYHA stage III to IV heart failure.

The prevalence of inter-VD was 47.5% in our study. Schuster *et al*. reported a prevalence of 45% for inter-VD in DCM.^9^ This prevalence was higher than in others series. Bader^10^ reported a prevalence of 23% in a population optimally treated for stable heart failure. This difference may be explained by the stage of heart failure. In the CARE-HF study, the prevalence of inter-VD among patients recruited by the conventional criteria of cardiac resynchronisation therapy was 62%.^8^

In our study the exclusive cause of wide QRS was LBBB. A number of patients (12) in our study population with normal QRS duration showed inter-VD. A greater proportion of patients with inter-VD was observed in group 1 (QRS ≥ 120 ms) compared to patients in group 2 (*p* = 0.03) (Table 4). This is in accordance with other series of patients.^6^,^8^,^10^,^11^

**Table 4.** Relationship Between QRS Duration And Interventricular Dyssynchrony (*p* = 0.03)

	*Group 1 (QRS ≥ 120 ms)*	*Group 2 (QRS < 120 ms)*	*Total*
Patients with inter-VD	7	12	19
Patients without inter-VD	1	20	21
Total	8	32	40

The IVMD was not significantly related to QRS duration even if there was a trend of increasing importance of IVMD according to QRS width (*p* = 0.06). Data in the literature showed a good correlation between the IVMD and the width of the QRS.^6^,^10^-^12^ This correlation improved slightly when patients with pulmonary hypertension or right ventricular dysfunction were excluded.^12^

We have shown a prevalence of 70% of intra-LVD, which seemed to be the most frequent type of mechanical dyssynchrony, irrespective of the QRS width (Table 3). Similar results were observed in other studies.^6^,^10^ Hospitalisation for heart failure is a predictor of the existence of left ventricular dyssynchrony and is significantly correlated with a high risk of early cardiac decompensation.^10^ There was a substantial proportion of patients with normal QRS duration who also showed intra-LVD (21/32) (Table 3). These findings point to the fact that the QRS width is not an accurate predictor of mechanical dyssynchrony.

Inter-VD is the principal factor associated with contractile impairment and is affected by CRT.^1^ The evaluation of dyssynchrony is done with CRT.

We noted that 21 patients with narrow QRS presented with intra-LVD. They could potentially have benefitted from CRT but if we rely on conventional criteria,^13^ these patients would have been excluded because they did not exhibit electrical dyssynchrony (defined by a QRS duration > 120 ms). Perez de Isla *et al*.^14^ noted that 38.4% of patients with QRS < 120 ms presented with intra-LVD. Ghio *et al*.^12^ found 29.5% of patients with QRS < 120 ms had left ventricular dyssynchrony. This prevalence was 72% in the study by Jurcut *et al*.^6^

In our study, in patients with intra-LVD, seven had an ESD ≥ 100 ms. Three of these seven had a QRS duration ≥ 120 ms. This cut-off value of 100 ms is an indicator of a good response to CRT.^1^ Improvement of left ventricular function after CRT is predicted by tissue Doppler imaging echocardiography. Penicka *et al*.,^15^ using a composite index of inter-VD and intra-LVD longer than 100 ms, achieved 88% accuracy in identifying patients who responded to CRT.

There were some study limitations. This study was performed on a consecutive sample of patients (40) who presented in our department with DCM in NYHA stage III to IV during a seven-month period. A larger study population would bring more significance to the present data. We were not able to determine the aetiology of the DCM in some patients.

## Conclusion

The assessment of cardiac dyssynchrony is possible in our country but it remains largely untapped due to the low availability of the techniques and the inaccessibility of CRT. Intra-ventricular mechanical dyssynchrony has a high prevalence in patients with DCM, irrespective of the QRS width. A substantial proportion of patients with DCM and narrow QRS exhibiting intra-LVD may benefit from CRT. These data emphasise the value of echocardiography in the screening of patients.
